# Filter Plating Method for Rendering Picocyanobacteria Cultures Free of Heterotrophic Bacterial Contaminants and Clonal

**DOI:** 10.3389/fmicb.2022.821803

**Published:** 2022-02-16

**Authors:** Sean M. Kearney, Allison Coe, Kurt G. Castro, Sallie W. Chisholm

**Affiliations:** Department of Civil and Environmental Engineering, Massachusetts Institute of Technology, Cambridge, MA, United States

**Keywords:** *Synechococcus*, *Prochlorococcus*, axenicity, clonality, plating

## Abstract

Isolates of the marine picocyanobacteria, *Prochlorococcus* and *Synechococcus*, are often accompanied by diverse heterotrophic “contaminating” bacteria, which can act as confounding variables in otherwise controlled experiments. Traditional microbiological methods for eliminating contaminants, such as direct streak-plating, are often unsuccessful with this particular group of microorganisms. While they will grow in pour plates, colonies often remain contaminated with heterotrophic bacteria that can migrate through the soft agar. Additionally, axenic clones of picocyanobacteria can be recovered *via* dilution-to-extinction in liquid medium, but the efficiency of recovery is low, often requiring large numbers of 96-well plates. Here, we detail a simple and effective protocol for rendering cultures of *Synechococcus* and *Prochlorococcus* strains free of bacterial contaminants while at the same time yielding clonal isolates. We build on the fact that co-culture with specific heterotrophs—“helper heterotrophs”—is often necessary to grow colonies of picocyanobacteria from single cells in agar. Suspecting that direct physical contact between the helper and the picocyanobacterial cells was not necessary for the “helper effect,” we developed a protocol in which the helper cells are embedded in soft agar pour plates, a filter overlaid on the surface, and a picocyanobacterial culture is diluted and then spotted on top of the filter. With this approach, motile contaminants cannot swim to the colonies, and it is possible to obtain the expected number of colonies from a given input (i.e., a Poisson distribution of colonies with an expected value equal to the input number of cells), thus ensuring clonal colonies. Using this protocol, we rendered three strains of *Synechococcus*, two strains of *Prochlorococcus*, and 19 new strains of *Synechococcus* from coastal seawater clonal and free of heterotrophic bacteria. The simplicity of this approach should expand the repertoire of axenic picocyanobacterial strains available for controlled physiological experiments. It will also enable the study of microdiversity in populations of picocyanobacteria by facilitating large-scale isolation of picocyanobacterial clones from a single source, including direct isolation from natural seawater.

## Introduction

Marine picocyanobacteria, which are at the base of marine microbial food webs, are frequently brought into culture using enrichments on nutrient-amended raw or filtered seawater, or by using dilution-to-extinction approaches (Waterbury and Stanier, 1981; Waterbury, 1986; Moore et al., 2002, 2007; Ahlgren and Rocap, 2006). However, these cultures are rarely (if ever) free of heterotrophic bacterial contaminants, due to the basal carbon in seawater and production of fixed organic carbon by the phototrophs that support these contaminants. Contaminant bacteria are present in the background of cultures used in many studies, even though detailed mechanistic studies of picocyanobacteria benefit from the removal of contaminating organisms. Heterotrophs can affect coarse-grained parameters like growth rate for *Prochlorococcus* (Morris et al., 2011), fine-grained parameters like gene expression (Biller et al., 2016), susceptibility to stressors (e.g., light, temperature, and pH; Ma et al., 2017), or interactions with other biotic factors (viruses or bacterivores), so obtaining contaminant-free cultures is key to advancing these picocyanobacteria as model systems (Aguilo-Ferretjans et al., 2021). However, they are often not purified out of culture because removing them is such a significant challenge.

In addition to rendering picocyanobacteria free of heterotroph contaminants, creating clonal cultures (derived from a single cell) presents an additional challenge. Cultures can be recovered from single cells by pour-plating (Brahamsha, 1996; Moore et al., 2007), cultivation on agar surfaces with specific helper heterotrophic bacteria, *sensu* Morris (Morris et al., 2008), or dilution-to-extinction (Moore et al., 2007; Berube et al., 2015). Typically, however, clonal cultures remain contaminated with heterotrophic bacteria—likely because heterotrophs can swim through soft agar toward the phototrophs.

One of the main reasons it is difficult to render picocyanobacterial cultures both clonal and axenic is that they have well-known metabolic interdependencies with their associated heterotrophs. For example, *Prochlorococcus* relies on heterotrophic bacteria to detoxify radical oxygen species, as it lacks the gene for the production of catalase (Morris et al., 2011, 2012). This dependency, as well as others (for example, recycling of nitrogen: López-Lozano et al., 2002; Zubkov et al., 2003; and phosphorous: Christie-Oleza et al., 2017), leads most of the cells that grow into robust cultures from a single cell using dilution-to-extinction to be those that are metabolically stabilized by the “contaminating” heterotrophs (examples of such stabilizing interactions abound: Sher et al., 2011; Aharonovich and Sher, 2016; Coe et al., 2016; Christie-Oleza et al., 2017; Ma et al., 2017; Biller et al., 2018; Roth-Rosenberg et al., 2020; Kearney et al., 2021). The function of these heterotrophs can sometimes be substituted by adding pyruvate to dilution-to-extinction cultures as an antioxidant, but pyruvate alone cannot completely replace the functions of heterotrophic bacteria (Moore et al., 2007; Coe et al., 2016). However, one challenge in using helper heterotrophic bacteria to facilitate robust growth is that they must be removed following recovery of clones.

Additionally, with dilution-to-extinction, recovery of clones is not quantitative (i.e., one cell in, one culture out), such that large numbers of 96-well plates are usually required to recover a small number of axenic clones, which poses another challenge to rendering marine picocyanobacteria clonal. Further, it is difficult to determine whether any resulting culture is truly clonal, that is, derived from a single cell from the onset, without quantitative recovery of input cells.

In the method that follows, we add a physical barrier between helper heterotrophs and picocyanobacteria, in order to take advantage of the benefits of co-culture with heterotrophic bacteria (Morris et al., 2008; Zborowsky and Lindell, 2019), and pour-plating techniques (Brahamsha, 1996; Moore et al., 2007; Laurenceau et al., 2020). This barrier preserves the helper function, as it is permeable enough to allow metabolic exchange, but eliminates the ability of contaminants to swim toward picocyanobacterial cells through soft agar. This method also ensures that these colonies are clonal, as it results in quantitative recovery of colonies from single cells on the surface of filters. The colonies can then be screened for the presence of contaminating heterotrophic bacteria. A large fraction (usually greater than 50%) will be axenic and can be selected for further study. We demonstrate the efficacy of this method for recovering axenic clonal cultures of a number of *Prochlorococcus* and *Synechococcus* strains already in culture and show that the approach can be used to isolate clones of *Synechococcus* directly from complex microbial communities in natural seawater.

## Materials and Equipment

### Equipment

-   Flow Cytometer (Luminex Guava easycyte 12HT Flowcytometer).
-   Light Meter (Walz Spherical Micro Quantum sensor US-SQS/L).
-   Environmental chamber, light, and temperature controlled (Percival Scientific Model I-36LLVL).
-   Water bath (ThermoScientific 5 L General Purpose Water Bath TSGP05).
-   Microwave (Panasonic Microwave Oven NN-SN966S).
-   Biosafety Cabinet (Labconco Purifier Logic+ Class II, Type A2).
-   Laminar Flow Hood (Tabletop Horizontal Laminar Flow Workstation, Envirco).
-   Epifluorescent Microscope (Zeiss Axioskop 2) (as an alternative to flow cytometry for cell counts).

### Materials

-   Pro99 Nutrient Stocks and Seawater Base [4].
-   BD Difco Marine Broth (MB 2216 Medium) (BD 279110).
-   Supor 47 mm 0.22 μm filters (Pall 60301).
-   Acrodisc Syringe Filters with Supor Membrane, Sterile-0.2 μm, 13 mm (Pall 4602).
-   BD Luer-Lok 1 ml syringes (BD 309628).
-   Petri plates, 100 mm (Fisher Brand FB0875713).
-   Sodium Sulfite (Sigma S4672).
-   Sodium Bicarbonate (Sigma S6014).
-   Parafilm.
-   BD Difco Agar Noble (BD 214220).
-   UltraPure Low Melting Point (LMP) Agarose (Invitrogen 16520050).
-   Sybr Green I Nucleic Acid Gel Stain-10,000X in DMSO (Thermo Fisher Scientific S7563).
-   TE Buffer pH 8.0 (Sigma 93283).
-   96-well tissue culture plates (Fisher Scientific FB012931).
-   Heterotrophic helper bacteria (*Alteromonas* str. EZ55; Morris et al., 2008), *Henriciella marinus* str. MIT2000 (this manuscript, isolated from *Prochlorococcus* str. MIT1214), or *Alteromonas* str. MIT1002 (Biller et al., 2015), or a heterotroph isolated from the original xenic culture.

## Methods

### Objective

The objective of the method detailed here is to obtain heterotrophic bacteria-free clonal cultures of *Synechococcus* or *Prochlorococcus* from xenic sources (cultures or raw seawater).

### Overview

**Figure 1:**
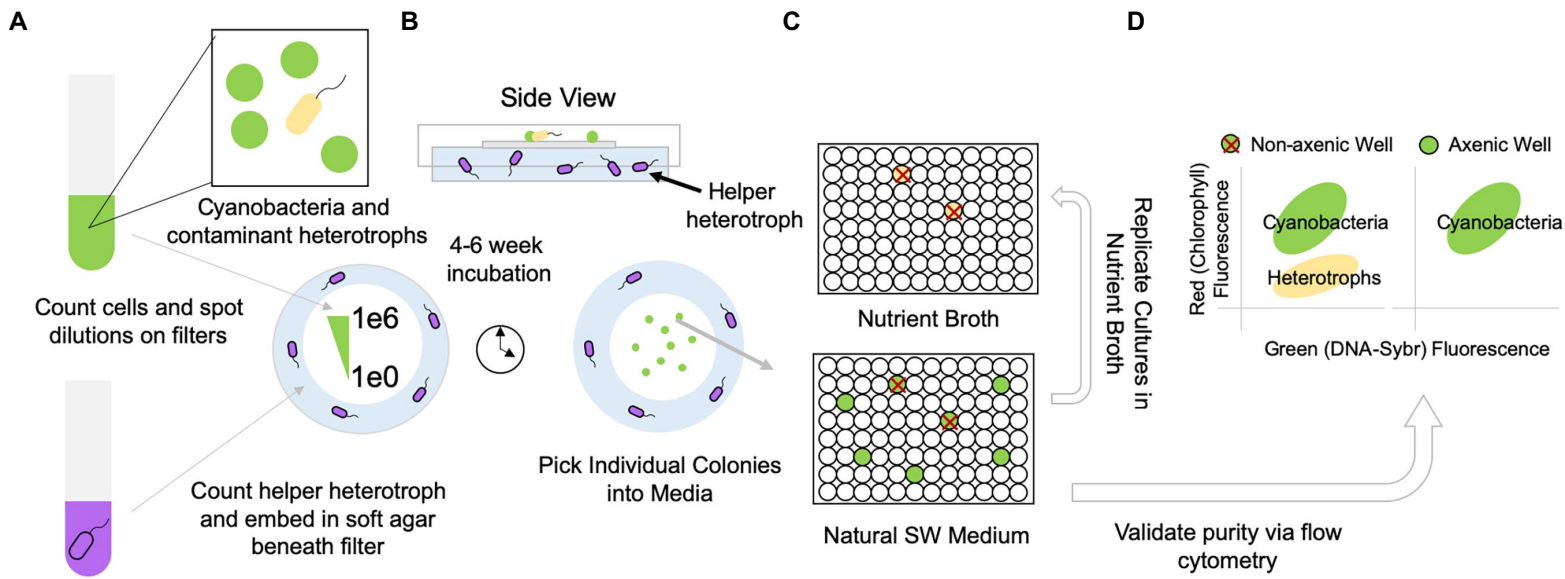
Workflow for obtaining contaminant (yellow)-free, clonal, isolates of *Synechococcus* and *Prochlorococcus* (green) by separating helper heterotrophs (purple) from picocyanobacteria using a filter on the surface of the helper cell lawn. **(A)** Cells of picocyanobacteria and contaminant heterotrophs as well as helper heterotrophs (purple) are counted *via* flow cytometry; the helper heterotrophs are embedded in a pour plate of cyanobacterial agar. Picocyanobacterial cultures are quantitatively diluted for spotting single cells on overlaid filter membranes. **(B)** After approximately 4–6 weeks (depending on the strain), colonies derived from single cells appear on filters and are picked into the original cell culture medium **(C)** plate is replicated (10% transfer) in nutrient broth to verify growth of any contaminant heterotrophs. **(D)** Cultures without contaminant heterotrophs are further verified *via* flow cytometry. Axenic cultures are transferred to larger volumes and purity is validated 1–2x more by flow cytometry.

**Figure 2:**
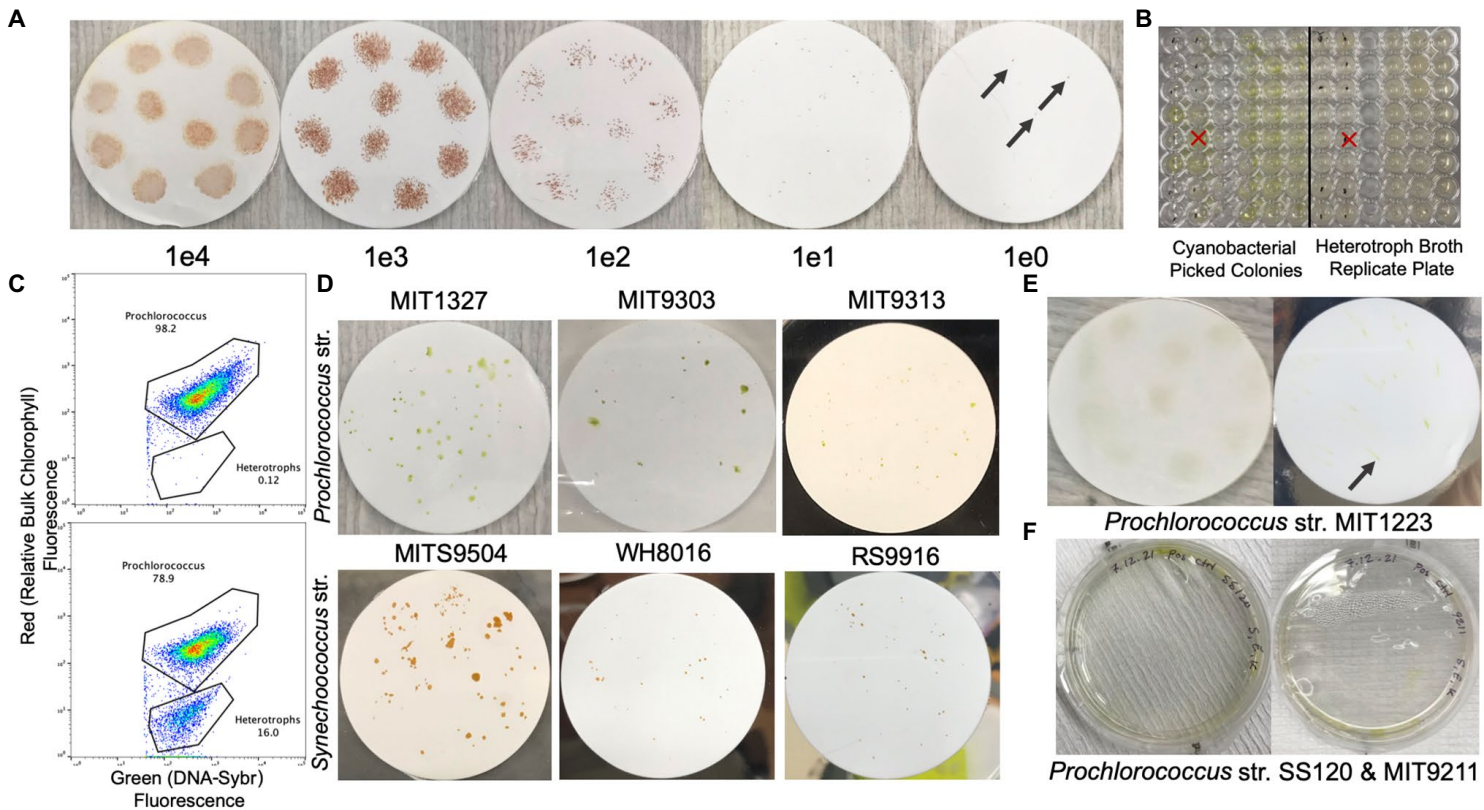
Filter plates with colonial growth of various *Prochlorococcus* and *Synechococcus* strains. **(A)** Dilution series of *Synechococcus* str. RS9916 on 0.2 μm Supor filters overlaid on natural seawater-based Pro99 with LMP agarose at 0.3%. Concentrations indicate cells per 10 μl spot on the filter (black arrows indicate pinpoint colonies). **(B)** Picocyanobacteria colonies picked into natural seawater-based Pro99 medium (left) and then replicated into MB 2216 Medium to screen for heterotrophs (right, dots on wells indicate contamination). **(C)** Example flow cytogram of a SYBR Green I DNA stained culture of axenic (top) and non-axenic (bottom) *Prochlorococcus*. **(D)** Examples of colonial growth of *Prochlorococcus* str. MIT1327, MIT9303, MIT9313, and *Synechococcus* str. MITS9504, WH8016, and RS9916. **(E)** Diffuse colonial growth of MIT1223 at 1e4 cells per spot (left), and 10 cells per spot (right, black arrow indicates pinpoint colony). **(F)** *Prochlorococcus* strains SS120 and MIT9211 with limited/patchy growth in pour plates embedded with helper heterotrophic bacteria.

We obtain single clones of *Synechococcus* or *Prochlorococcus* by diluting cultures onto filters overlaid on medium containing helper heterotrophs (Figures 1A, 2A). To optimize recovery of axenic clones, we pick as many individual colonies as possible from the highest dilution (1 cell/spot) into Pro99 natural seawater medium arrayed in 96 well plates (Figure 1B). We do a 10% replication of these wells into a 96-well plate containing heterotrophic growth medium (2216 Marine Broth), and after 2–3 days, mark wells on the original Pro99 plate that exhibit contamination of the replicated heterotrophic growth medium (Figures 1C, 2B). Picked colonies in Pro99 will be visibly green (in the case of *Prochlorococcus*) after 1–2 weeks. We screen a subset of the remaining clones that are free of apparent contamination based on growth in replicate heterotrophic medium by SYBR staining and flow cytometry (Figure 1D). If flow cytometry is unavailable, microscopic examination of SYBR strained cultures is also feasible. Clones free of heterotrophic contaminants lack a secondary population of cells with low red fluorescence (chlorophyll) and green fluorescence (SYBR), while contaminated clones typically have an apparent population in this region (Figures 1D, 2C).

### Protocol for Filter-Plate Recovery of Axenic Clones of *Synechococcus* and *Prochlorococcus*

1. One must first choose the strain of helper heterotroph to be employed. Using a strain isolated from the original xenic culture may work best but any well-established helper heterotroph such as *Alteromonas macleodii* str. EZ55 (Morris et al., 2008) can be used. Several days in advance of filter plating, recover a culture of the helper heterotrophic bacteria by streak-plating on agar (i.e., MB 2216). For both the *Alteromonas* and *Henriciella* strains used in this paper, a single colony from the streaked plate is transferred into MB 2216 broth and cultivated until mid-exponential phase (~24–48 h, but is strain-dependent) at 25°C (if available, shake at 250 RPM).
2. On the day of filter plating, obtain cell counts for both the picocyanobacteria and the helper heterotroph grown in MB 2216 from step 1 using a flow cytometer (see Figure 1). These cell counts will also confirm that contaminating heterotroph counts in the cyanobacterial culture are sufficiently low to proceed. Specifically, heterotrophs should be less than 50% (ideally less than 10%) of the total number of cells in the cyanobacterial culture to yield colonies free of heterotrophic bacteria. If the heterotrophic bacteria outnumber the picocyanobacteria, maintaining the cultures for several passages in exponential growth should reduce the number of heterotrophic contaminants. Alternatively, successive rounds of purification by this method could result in more robust outcomes. Indeed, to purify *Synechococcus* str. RS9916, we found that all clones obtained from one round of filter plating remained associated with heterotrophs, which were removed in a subsequent round.
  Diluted xenic cultures of *Synechococcus* or *Prochlorococcus* are prepared by diluting down to ~500 cells/μl (or appropriate dilution based on the flow cytometer restrictions) using 0.2 μm filtered seawater. To count cells, prepare a 30X concentrate of SYBR by adding 3 μl of 10,000X SYBR Green I stock to 1 ml 1x TE buffer (these stocks are stored at −20°C and will be viable for about a month). To the diluted cultures, the prepared 30X SYBR is added to reach a final concentration of 1.5X. After SYBR is added, cultures are incubated in the dark for 30–60 min, before counting on the flow cytometer. This process is repeated with the helper heterotroph culture to also obtain cell counts (Figure 1A).
  If a flow cytometer is not available for counting, one can use fluorescence microscopy to manually count cell concentrations in cultures of picocyanobacteria and SYBR-stained heterotrophic bacteria.
3. Prepare fresh stocks of 1 M sodium sulfite (used as a reductant; Brahamsha, 1996) and 6 mM sodium bicarbonate (as a buffer and source of carbon for photosynthesis; Moore et al., 2007) in Milli-Q water and filter sterilize using a 0.2-μm syringe filter.
  Using filtered and autoclaved natural seawater, agar plates are prepared as follows: (1) add 0.3% (w/v) noble agar or LMP agarose (UV-treated in the biosafety cabinet for 5–10 min) to ~100 ml or 20% of the final volume of seawater needed in a sterile glass container; (2) heat the agar in a microwave (using short durations to avoid overboiling) until it comes to a boil and dissolves; (3) cool the solution to 45°C in a water bath; (4) remove from the bath and aseptically add Pro99 nutrients, sodium sulfite (1 mM final concentration), and sodium bicarbonate (6 mM final concentration); (5) cool the medium to 35°C in a water bath; (6) add 1e5 cells/ml of the helper heterotrophic bacteria, gently swirl to mix, and immediately pour 15–20 ml of the agar medium per 100 mm petri dish; and (7) cool in sterile environment for at least 10 min.
4. After confirming that the contaminating heterotrophs in the xenic culture are sufficiently low to proceed (again, ideally less than 10% of cells in the culture), prepare 10-fold serial dilutions in 1 ml seawater of the xenic culture, aiming for 1e6, 1e3, 5e2, 2.5e2, and 1.25e2 cells/ml. After cells are spotted onto the filter, this will result in, on average, 1e5, 1e2, 5, 2.5, and 1.25 cells per spot (Figures 1A, 2A).
5. Using ethanol or flame-sterilized tweezers, transfer a 47 mm 0.2 um Supor filter (non-sterile filters are fine, we leave it to the reader to experiment with other filter types) to the surface of the cooled and semi-solidified agar and immediately spot the lowest concentration of the xenic culture (1.25e2 cells/ml) by pipetting twelve 10 μl spots on the surface of the filter (Figure 1B). Allow the open plates to dry in a laminar flow hood for 30–60 min (spots must dry completely; otherwise heterotrophic bacteria can potentially swim to the picocyanobacteria or grow within the spots). Do not place filters on the plates in advance or the drying process will take considerably longer. Cover the plate and seal with parafilm. Repeat process using one or more of the higher concentrated dilutions (1e6–1e4), which serve as positive controls for growth.
6. Incubate the plates in the environmental chamber at the light level (obtained with a light meter) and temperature of the original xenic culture. The plates should be kept upright (filter side up) in a sealed container (i.e., plastic bag). It is important not to put the plates directly over a heat source, like a light bulb, as it will generate a temperature gradient and cause condensation. This issue can be avoided by placing plates near fans or on top of elevated surfaces (i.e., test tube rack) so that the convective medium is air rather than a solid surface separated from the light bulb. If there is no growth after 1–2 weeks for the highest cell concentrations, it is likely that the experiment failed.
7. Once individual colonies are visible on the filters, use a sterile pipette tip (i.e., 10 μl tip), loop, or pick to transfer individual colonies into pre-loaded 200 μl Pro99 medium in a tissue culture-treated 96-well plate. Pipette tips can be pre-loaded with 1 μl sterile seawater to provide capillary action to draw up the colony. After picking all the colonies into Pro99 medium, replicate the 96-well plate by doing a 10% transfer (20 μl of the Pro99) of the culture volume into 180 μl of sterile Difco MB 2216 Broth to assess heterotrophic contamination of the original colony. This plate will serve as a purity screen for the individual colonies (Figures 1C, 2B).
8. If wells in the plate containing MB 2216 broth appear opaque, this indicates heterotrophic growth. The lid of the plates above contaminated wells should be marked to indicate they will not produce axenic cultures (Figures 1C, 2B). After doing so, identify remaining uncontaminated wells that have produced growth (in the case of *Prochlorococcus*, green wells) and confirm purity using flow cytometry as described in step 2. Transfer all axenic wells into larger volumes of Pro99 medium (i.e., ≥3 ml) and confirm axenicity at least 1–2 more times after cells have reached exponential phase. Sometimes low-level contaminants will become visible in larger volumes of culture (Figures 1D, 2C).
9. For picocyanobacterial isolates with known genomes, resequencing provides a facile method for identifying genomic heterogeneity in clones obtained by this method. If isolates are obtained from seawater with this method, clones can be differentiated by sequencing of the 16S-23S internal transcribed spacer region, *petB* gene [3, 11, 12] or whole genome sequencing.

## Results

The filter plating protocol described here will produce individually arrayed clones of picocyanobacteria that are free of heterotrophic bacterial contaminants after a series of plating techniques involving helper bacteria, and downstream verifying techniques (Figure 1). Because this method has two purposes: (1) to remove heterotrophic contaminants and (2) to obtain clones of picocyanobacteria, either outcome can be considered a success depending on initial conditions. It is possible for clones to be contaminated with heterotrophic bacteria (i.e., if a single colony still contains heterotrophs after filter plating), but the remaining heterotroph(s) can typically be removed by a secondary round of purification *via* filter plating.

### Strain-Dependent Outcomes

Using this method, we recovered axenic clones from three strains in the *Prochlorococcus* LLIV clade and one strain (MIT1223) in a separate low light grouping, as well as four *Synechococcus* strains from three distinct clades (Table 1). All filter plating attempts (except for *Prochlorococcus* str. MIT1327, for which we used *Alteromonas macleodii* str. MIT1002 as the helper) were done with a single helper heterotroph, *Henriciella pelagia*, chosen because we observed that it consistently yielded colonies from single cells when plated with *Synechococcus* str. RS9916 (Figure 2A; Table 1). While we were able to render six strains clonal and axenic with this method (Figure 2D), we were also able to obtain colonies and clones (non-axenic) of *Prochlorococcus* str. MIT1223 (Figure 2E; Table 1) and *Synechococcus* str. MITS9509 (Table 1). An additional round of filter plating on these strains would likely yield axenic colonies.

**Table 1:** Recovery, clonality, and axenicity of clones of *Synechococcus* and *Prochlorococcus* following filter plating with *Henriciella pelagia* str. MIT2000, except for *Prochlorococcus* str. MIT1327, which was recovered with *Alteromonas macleodii* str. MIT1002.

	Axenic at start?	Any growth on filters?	Clonal?	Axenic and clonal?
***Synechococcus***				
str. RS9916	No	Yes	Yes	Yes
str. MITS9504	No	Yes	Yes	Yes
str. WH8016	No	Yes	Yes	Yes
str. MITS9509	No	Yes	Yes	No
***Prochlorococcus***				
str. MIT9313	Yes	Yes	Yes	Yes
str. MIT9303	No	Yes	Yes	Yes
str. MIT1327	No	Yes	Yes	Yes
str. MIT9312	Yes	Yes	No	N/A
str. MED4	Yes	No	No	N/A
str. NATL2A	Yes	No	No	N/A
str. MIT1223	No	Yes	Yes	No
str. SS120	No	No	No	N/A
str. MIT9211	No	No	No	N/A

Clonal cultures had 100% plating efficiency.

While attempts with the above strains yielded clonal and axenic colonies, in single attempts to obtain colonies for other strains, we sometimes observed limited or no growth on filters. Therefore, it is suggested that the preliminary check for success of the method is observable growth on filters with undiluted samples. Within 1–2 weeks (or sometimes less), these spots should grow dense enough to be visible. Then, we typically plate a dilution series of the spots to confirm that lower concentrations are likely to develop visible colonies as well (Figure 2A). However, in some cases, even though the undiluted spot of a sample will grow on the filter, subsequent dilutions do not. At this point, alternative conditions should be tested (for instance, using an alternative helper heterotroph). For example, we observed this tendency with a culture of *Prochlorococcus* strain MIT9312, which grew undiluted on filters, but did not grow when spotted on filters at 1e5 cells/spot or lower (Table 1). Similarly, *Synechococcus* str. MITS9509 grew in single colonies at dilutions down to 1e2 cells/spot, but all clones picked at this dilution remained contaminated with heterotrophs, suggesting that a more bespoke helper heterotroph selection would permit axenic growth.

By contrast, however, we could never obtain growth on filters or even robust growth in pour plates of *Prochlorococcus* str. SS120 and str. MIT9211 (Figure 2F), suggesting that other factors may limit their ability to grow on surfaces. This limitation was also shown in previous work with SS120, in which no colonies were obtained in any helper heterotroph-pair pour-plating [7]. Thus, we highly recommend the use of controls (direct pour plates and undiluted spotting) to enable quicker experimental iteration when attempting this method.

### Isolating Colonies of Picocyanobacteria From Natural Seawater

**Figure 3:**
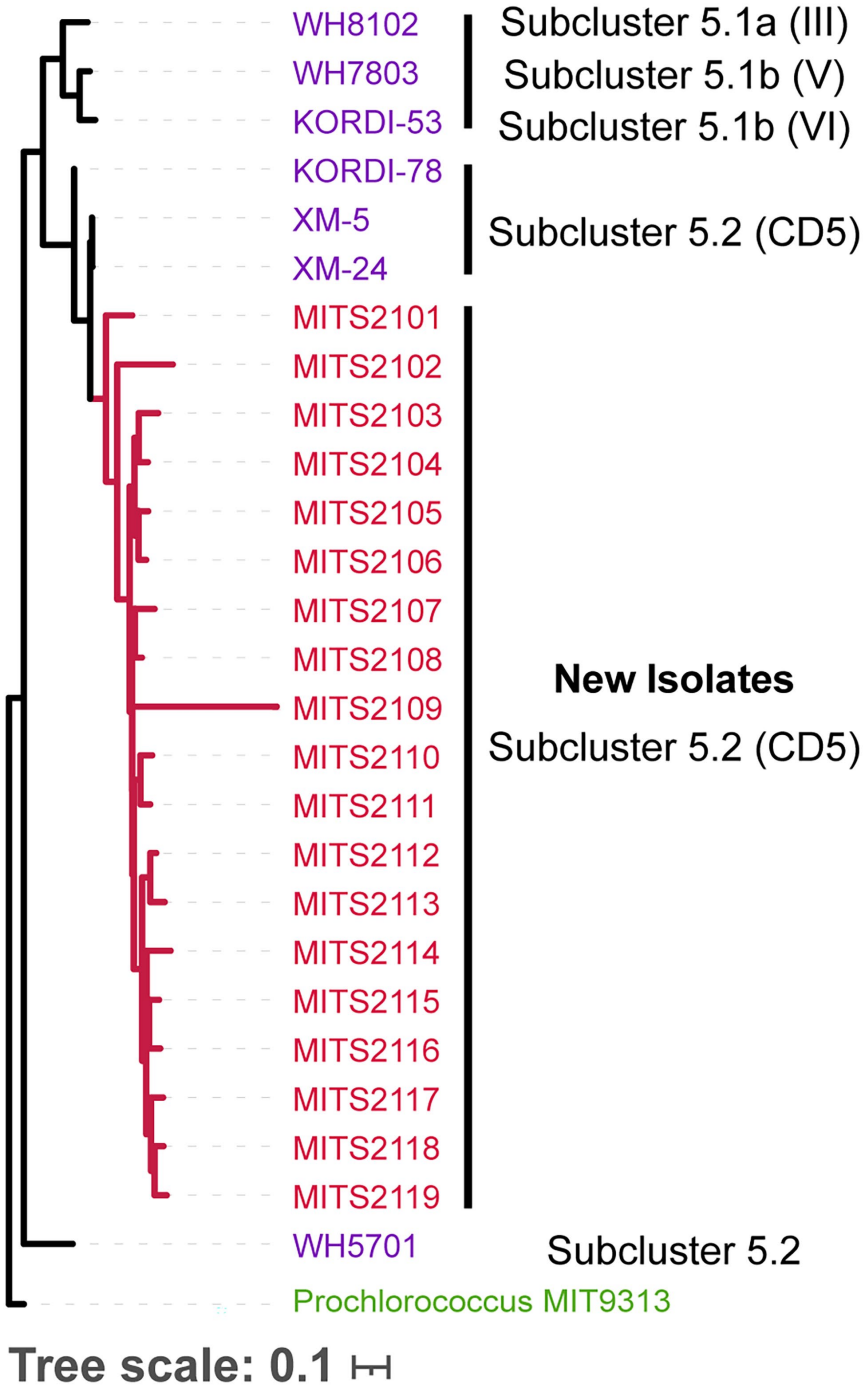
Phylogenetic tree of the 16S-18S internal transcribed spacer (ITS) sequence (for molecular methods, see Rocap et al., 2002; MUSCLE Edgar, 2004 was used to generate the ITS alignment, and FastTree Price et al., 2010 to generate the tree, sequences are available at https://doi.org/10.6084/m9.figshare.17076446) for *Synechococcus* isolates obtained from natural seawater using the filter plating technique described here (red), with previously isolated *Synechococcus* strains (purple), annotated with their lineages. Strains are colored by subcluster. *Prochlorococcus* str. MIT9313 ITS (green) sequence serves as the outgroup to root the tree.

In addition to cloning and purifying existing cultures of *Prochlorococcus* and *Synechococcus*, we could also directly isolate colonies of marine *Synechococcus* from coastal seawater. More importantly, the approach resulted in the isolation of novel strains of *Synechococcus*, which likely belong to *Synechococcus* clade CB5 in subcluster 5.2 (Figure 3). Briefly, we followed the protocol as above, but instead of spotting diluted culture on filters, we spotted 10 μl aliquots of 0.8 μm filtered coastal seawater from Woods Hole, MA. After 2–3 weeks, red colonies of *Synechococcus* (identified by flow cytometry) became visible on filters, and we selected 19 of these for amplification and sequencing of the ITS region (Figure 3). This approach has not been tested on open ocean seawater, but we suspect will have promise for obtaining strains of *Prochlorococcus* and *Synechococcus* from such environments.

## Conclusion and Future Directions

The method presented here provides an alternative to existing methods—particularly for strains recalcitrant to recovery by dilution-to-extinction or pour-plating—by combining dilution plating on filters with the recovery function of helper heterotrophic bacteria. Recovery of colonies on filters likely depends on the selection of the helper heterotroph, as it does for direct plating [7]. The approach eliminates the possibility of mobile contaminants swimming into colonies and, in our hands, enables quantitative yield of clonal axenic isolates. While not a perfect substitute for dilution-to-extinction, we have used this method successfully in cases where dilution-to-extinction has failed. It has the added benefit of being less labor and resource intensive.

As we have described above, the method did not work for all strains. We anticipate that for the strains of *Prochlorococcus* that can grow on pour plates, but were unable to grow on filters (i.e., MED4 and NATL2A), an alternative helper heterotroph, or a mix of pre-cultured heterotrophs—ideally strains that originated from the xenic culture—would enable growth on filters. Alternatively, if heterotroph isolates are unavailable, embedding the xenic culture directly in the soft agar may enable growth on filters. This strategy would retain existing metabolic dependencies in the cultures.

As seen in the examples of *Prochlorococcus* str. MIT9211 and SS120, some cultures are unable to grow robustly even in soft agar pour plates, suggesting that growth in (or on) semi-solid medium (let alone a filter) may not be possible for these strains. In these cases, one might try to render them monoxenic by pre-seeding 96-well arrayed liquid seawater-based medium with helper heterotrophic bacteria and then diluting single cells of *Prochlorococcus* into these wells. Helper heterotrophs could subsequently be removed through differential antibiotic susceptibility (Morris et al., 2008) from the picocyanobacteria or by raising a phage against the helper heterotroph. In the spirit of the filter-plating method, commercial (but costly) 96-well transwell plates, in which an apical insert with a 0.4-um pore size is placed in a basal medium, could be used to enable physical separation of single cells of picocyanobacteria on the apical side from helper heterotrophs on the basal side.

We anticipate that this method will be useful for a variety of researchers studying picocyanobacteria and other marine or aquatic photosynthetic organisms which frequently associate in culture with heterotrophic bacteria. We present initial success in rendering three strains of *Synechococcus* and three strains of *Prochlorococcus* clonal and axenic, as well as direct isolation of *Synechococcus* clones from coastal seawater. Further isolation of novel strains of picocyanobacteria may be possible using this method, particularly when co-culture can facilitate interactions that would otherwise prevent cultivation of strains.

Ironically, the problem of contaminated cultures had its solution embedded within: leveraging the metabolic dependencies of picocyanobacteria on their heterotrophic partners was what allowed us to remove these “contaminants” and work toward a synthetic approach to microbial ecology, with the goal of understanding how interactions stabilize and contribute to the resilience of microbial ecosystems. Continuing to capitalize on the inherent complexity of natural communities promises to transform their understanding.

## Data Availability Statement

The datasets presented in this study can be found in online repositories. The names of the repository/repositories and accession number(s) can be found at: https://figshare.com/, https://figshare.com/articles/dataset/FilterSyn_fasta/17076446.

## Author Contributions

SK, AC, and SC: conceptualization. SK, KC, and AC: methodology, validation, and investigation. SK: formal analysis, data curation, writing-original draft, visualization, and project administration. SK and SC: resources and funding acquisition. SK, KC, AC, and SC: writing-review and editing. SK and AC: supervision. All authors contributed to the article and approved the submitted version.

## Funding

This work was funded by grants from the Simons Foundation (Life Sciences Project Award ID 337262, SC; SCOPE Award ID 329108, SC). SK is funded by the Simons Foundation Fellowship in Marine Microbial Ecology (SCOPE Award ID 649394).

## Conflict of Interest

The authors declare that the research was conducted in the absence of any commercial or financial relationships that could be construed as a potential conflict of interest.

## Publisher’s Note

All claims expressed in this article are solely those of the authors and do not necessarily represent those of their affiliated organizations, or those of the publisher, the editors and the reviewers. Any product that may be evaluated in this article, or claim that may be made by its manufacturer, is not guaranteed or endorsed by the publisher.
